# TRIP13 identified by WGCNA as a progression-related and prognostic biomarker in ovarian cancer

**DOI:** 10.3389/fmed.2026.1765576

**Published:** 2026-03-12

**Authors:** Hanyue Zhang, Moru Zhang, Yanyu Hu, Ying He

**Affiliations:** Department of Gynecology, The First Affiliated Hospital of Hebei North University, Zhangjiakou, China

**Keywords:** CENPF, ovarian cancer, therapeutic target, TRIP13, WGCNA

## Abstract

Ovarian cancer (OC) remains one of the most lethal gynecological malignancies, and its molecular mechanisms are still not fully elucidated. Although TRIP13 has been linked to several cancers, its role in OC is not clearly defined. This study aimed to identify TRIP13 as an OC-related gene and evaluate its clinical significance through integrated bioinformatic analyses and experimental validation. Gene expression datasets GSE81778 and GSE140082 were retrieved from GEO. Differentially expressed genes (DEGs) between OC and benign ovarian tissues were identified using the “limma” package. GO and KEGG analyses were conducted for functional enrichment. Hub genes were screened using the MCODE plugin in Cytoscape. Weighted gene co-expression network analysis (WGCNA) was applied to identify clinically relevant modules, leading to the selection of TRIP13 and CENPF. TRIP13 protein expression was confirmed by immunohistochemistry in OC and normal ovarian tissues. Clinical data were analyzed to determine the association between TRIP13 expression and patient survival (PFS). A total of 532 DEGs were identified. MCODE screening yielded 25 hub genes, while WGCNA revealed 19 gene modules, with the magenta module significantly associated with OC traits. TRIP13 was markedly overexpressed in OC tissues and positively correlated with higher tumor stage. Kaplan–Meier and multivariate Cox analyses in our independent clinical validation cohort demonstrated that high TRIP13 expression was an independent predictor of poorer progression-free survival. TRIP13 is identified as a potential oncogenic gene in ovarian cancer. Its high expression and association with advanced disease highlight its value as a biomarker and potential therapeutic target.

## 1 Introduction

Ovarian cancer is one of the most common and lethal malignancies of the female reproductive system. Although it can occur at any age, its global incidence has shown an upward trend over recent decades (1, 2). According to Global Cancer Statistics 2022, an estimated 324,398 new cases and 206,839 deaths occurred worldwide, underscoring the substantial global health burden posed by ovarian cancer (3). Due to the absence of specific symptoms and the limited efficacy of current screening strategies, early diagnosis remains challenging, and the majority of patients present with advanced-stage disease at the time of treatment (4).

Although significant advances have been made in cytoreductive surgery, platinum-based chemotherapy, and maintenance therapies, including PARP inhibitors, most patients eventually experience disease recurrence and develop platinum-resistant disease, resulting in poor overall prognosis (5). The underlying etiology and molecular mechanisms of ovarian cancer remain incompletely understood (6), although a variety of factors—including genetic predisposition, gynecologic conditions, reproductive factors, chromosomal abnormalities, and gene alterations—have been implicated. Therefore, there is a critical need to identify novel biomarkers and therapeutic targets, which may provide insight into disease pathogenesis and guide the development of more effective diagnostic and treatment strategies.

With the rapid development of high-throughput sequencing technologies, gene expression profiles and microarray datasets have been widely applied to identify differentially expressed genes (DEGs) in various malignancies (7). Systematic analysis of these datasets has proven to be a powerful approach for generating novel biological insights into ovarian cancer (OC). Numerous studies have demonstrated that integrative bioinformatics analyses offer reliable and effective strategies for identifying candidate biomarkers and elucidating the molecular mechanisms underlying tumor initiation and progression.

Weighted gene co-expression network analysis (WGCNA) is an unsupervised systems biology method used to identify modules of highly co-expressed genes and to explore their associations with clinical phenotypes (8). This approach facilitates the detection of key regulatory genes within biologically relevant networks, enabling the identification of potential drivers of disease (9). WGCNA has been extensively used across multiple disease contexts, including cancer, to uncover critical molecular signatures and therapeutic targets, demonstrating significant value in advancing mechanistic understanding and improving clinical outcomes (10).

In this study, two gene expression datasets (GSE81778 and GSE140082) were retrieved from the Gene Expression Omnibus (GEO) database. Initially, differentially expressed genes (DEGs) were identified from GSE81778, followed by functional enrichment analyses and protein–protein interaction (PPI) network construction to explore potential molecular mechanisms involved in ovarian cancer carcinogenesis and progression. Subsequently, weighted gene co-expression network analysis (WGCNA) was performed on the GSE140082 dataset to identify clinically relevant co-expression modules and extract the corresponding gene sets. By intersecting the module-derived genes with the PPI-based candidates, two central hub genes—TRIP13 and CENPF—were identified. Given the limited evidence regarding the role of TRIP13 in ovarian cancer, further experimental validations, including immunohistochemistry and patient survival analysis, were conducted to confirm its differential expression at the protein levels and to evaluate its prognostic significance. Among the two candidates, TRIP13 was selected for extensive experimental validation due to its less defined role in ovarian cancer compared to CENPF and its strong preliminary correlation with advanced disease traits in our analysis.

## 2 Methods

### 2.1 Data acquisition and preparation

Two ovarian cancer-related gene expression datasets (GSE81778 and GSE140082) were obtained from the Gene Expression Omnibus (GEO) database. The GSE81778 dataset contains 5 benign ovarian tumor samples and 19 epithelial ovarian cancer (EOC) samples. The GSE140082 dataset includes 379 ovarian cancer samples; among them, 276 EOC samples with complete clinical annotations were selected for subsequent analysis. To assess sample distribution patterns, detect potential outliers, and evaluate batch effects, principal component analysis (PCA) was performed on the normalized expression matrices.

### 2.2 Identification of differentially expressed genes between benign ovarian tumor tissues and EOC tissues

Differential expression analysis for the GSE81778 microarray dataset was conducted using the Limma package in R. After data normalization, DEGs between benign ovarian tumor tissues and epithelial ovarian cancer (EOC) tissues were identified based on the criteria of *p* < 0.05 and |log₂ fold change| ≥1.5. A heatmap was generated to visualize the expression patterns of the identified DEGs across the two groups.

### 2.3 GO and KEGG enrichment analysis of DEGs

Gene Ontology (GO) enrichment analysis, including biological process (BP), molecular function (MF), and cellular component (CC) categories, was performed to investigate the functional roles of the identified DEGs. Kyoto Encyclopedia of Genes and Genomes (KEGG) pathway analysis was further conducted to explore the potential signaling pathways involved in ovarian cancer development. GO annotations from the org.Hs.eg.db database were used as the background set, and enrichment analysis was carried out using the cluster Profiler package in R. The same tool was applied to KEGG pathway enrichment analysis, with pathway annotations obtained through the KEGG REST API. Gene sets with a size between 5 and 5,000 were included, and pathways with *p* < 0.05 and false discovery rate (FDR) <0.25 were considered statistically significant.

### 2.4 Weighted gene co-expression network analysis

The GSE140082 dataset was preprocessed by removing the 50% of genes with lowest variance and filtering outlier genes and samples using the good SamplesGenes function in WGCNA. A scale-free co-expression network was constructed by calculating Pearson correlations and generating a weighted adjacency matrix with a soft-thresholding power of 3. The adjacency matrix was transformed into a topological overlap matrix (TOM) to assess network connectivity. Genes were clustered into co-expression modules via hierarchical clustering with a minimum module size of 30, and modules with eigengene dissimilarity <0.25 were merged, yielding 19 modules. The grey module included unassigned genes. Module membership (MM) and gene significance (GS) were calculated, and genes with |MM| >0.8 and |GS| >0.1 were considered hub genes.

### 2.5 Single-gene set enrichment analysis

GSEA software (version 3.0; http://software.broadinstitute.org/gsea/index.jsp) was used for precise pathway enrichment analysis. Samples were stratified into high-expression (upper 50%) and low-expression (lower 50%) groups based on the expression level of the gene of interest. The “c2.cp.kegg.v7.4.symbols.gmt” gene set, downloaded from the Molecular Signatures Database was used to assess KEGG pathway enrichment associated with the gene. Default GSEA parameters were applied unless otherwise specified.

### 2.6 TCGA database exploration

Gene expression profiling interactive analysis (GEPIA, http://gepia.cancer-pku.cn/) is an interactive web server for analyzing gene expression based on data from The Cancer Genome Atlas (TCGA). Owing to its large sample size, GEPIA provides reliable results for gene expression studies. In this study, GEPIA was employed to perform subsequent analyses, including differential expression and survival analysis of genes of interest.

### 2.7 Immunohistochemical (IHC) verification of TRIP13 expression

Formalin-fixed paraffin-embedded (FFPE) tissue sections (4 μm) from 70 patients with high-grade serous ovarian cancer (HGSOC) and 34 normal ovarian controls were deparaffinized in xylene and rehydrated through graded alcohols. Heat-induced antigen retrieval was performed in citrate buffer, followed by cooling to room temperature. Endogenous peroxidase activity was blocked with 3% H_2_O_2_ for 5 min, and nonspecific binding was blocked with 5% goat serum for 15 min. Sections were incubated with a primary antibody against TRIP13 (Catalog: PA5-52193; Company: Thermo Fisher Scientific; Dilution: 1:200) overnight at 4 °C, followed by horseradish peroxidase (HRP)-conjugated secondary antibody (Catalog: G1213-100UL; Company: Sevill; Dilution: 1:200) for 1 h at room temperature. Visualization was achieved using DAB, and nuclei were counterstained with hematoxylin.

### 2.8 Evaluation method

Two independent pathologists, blinded to clinical data, scored the slides. Staining intensity was graded as 0 (negative), 1 (light yellow), 2 (brownish-yellow), or 3 (dark brown), and the proportion of positive tumor cells was categorized as 0 (0%), 1 (0–25%), 2 (25–50%), 3 (50–75%), or 4 (≥75%). The final IHC score (0–12) was calculated by multiplying intensity and proportion scores. High expression was defined as a score ≥6. Inter-observer agreement was assessed using Cohen’s kappa coefficient (*κ* = 0.82).

### 2.9 Survival follow-up and prognostic analysis

Overall survival (OS) was defined as the interval from diagnosis to death or last follow-up, and progression-free survival (PFS) was defined as the interval from the date of surgery to tumor recurrence or death. Patients were stratified into high- and low-TRIP13 expression groups based on IHC scores. Kaplan–Meier survival curves were generated, and differences between groups were assessed using the log-rank test. Multivariate Cox proportional hazards regression models, adjusting for age and FIGO stage, were employed to identify independent prognostic factors, with hazard ratios (HRs) and 95% confidence intervals (CIs) reported.

## 3 Results

### 3.1 Systematic screening of differentially expressed genes and functional enrichment analysis

**Figure 1:**
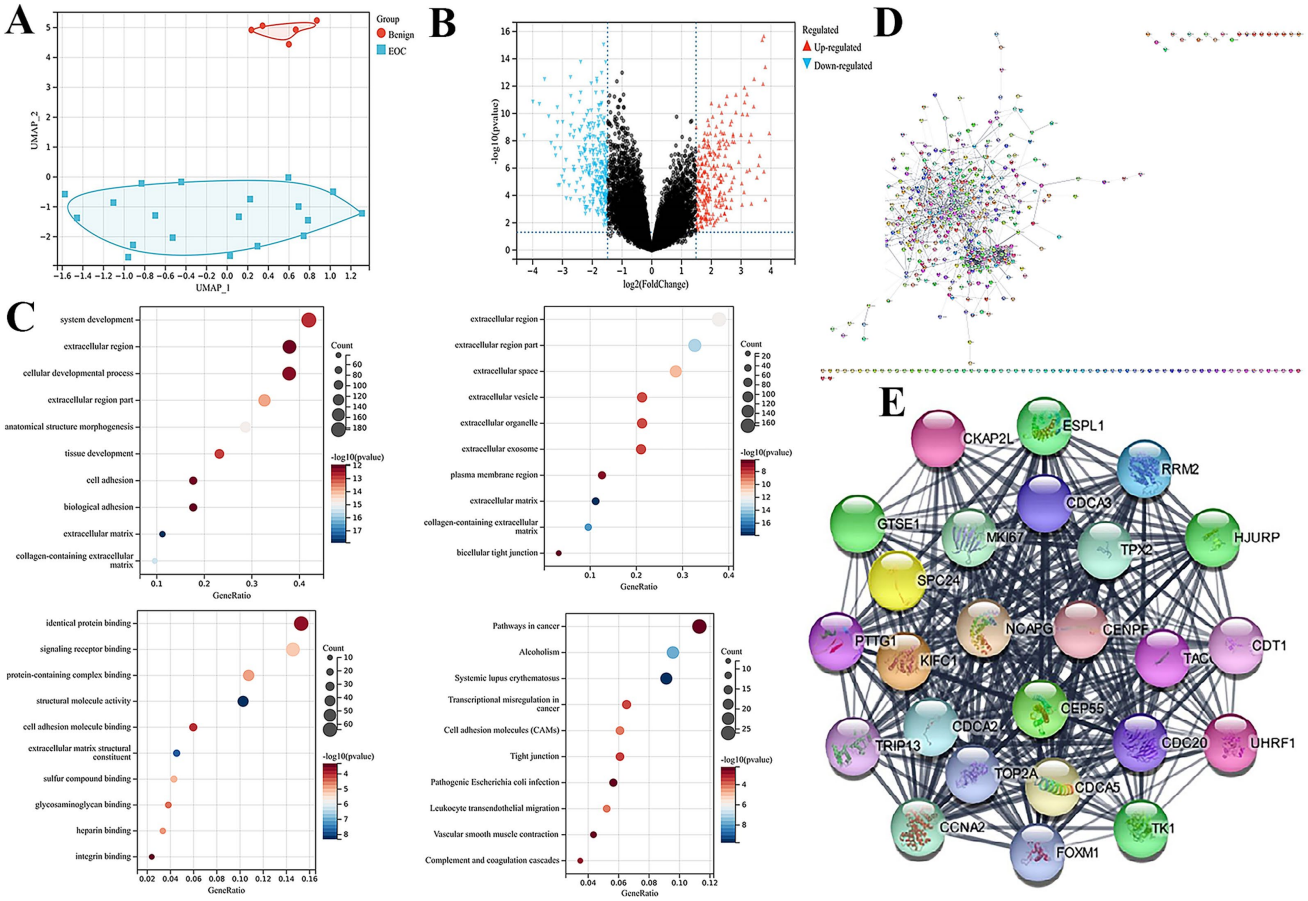
Systematic screening of differentially expressed genes and functional enrichment analysis. **(A)** Principal component analysis (PCA) showing distinct separation between experimental groups in the GSE81778 dataset. **(B)** Volcano plot displaying the results of differential expression analysis, identifying statistically significant differentially expressed genes (DEGs). **(C)** Functional enrichment analysis of the DEGs. Gene Ontology (GO) biological process, GO cellular component, GO molecular function, and KEGG pathway enrichment terms. **(D,E)** Protein–protein interaction network of the DEGs was constructed and analyzed using Cytoscape to identify key hub genes, shown from different visual perspectives.

In the GSE81778 dataset, principal component analysis (PCA) revealed a distinct separation between benign ovarian tumor tissues and epithelial ovarian cancer (EOC) tissues based on global transcriptomic patterns, indicating substantial biological heterogeneity within the cohort (Figure 1A). Differential expression analysis identified 532 differentially expressed genes (DEGs), including 258 upregulated and 274 downregulated genes (Figure 1B).

Gene Ontology (GO) enrichment analysis showed that these DEGs participate in several cancer-related biological processes, particularly those associated with organismal and cellular developmental regulation, cell differentiation, and multicellular organismal processes, which are frequently dysregulated during tumor initiation and progression. At the cellular component level, the DEGs were predominantly localized to the extracellular region, extracellular vesicles, and associated organelles, consistent with their potential involvement in intercellular communication and modulation of the tumor microenvironment. In terms of molecular function, significant enrichment was observed in protein binding, receptor binding, and adhesion molecule interactions, highlighting their role in multiple regulatory signaling axes (Figure 1C).

KEGG pathway enrichment further demonstrated that the DEGs were associated with cancer-related pathways, transcriptional dysregulation in cancer, and cell adhesion molecule (CAMs) pathways. Enrichment in the systemic lupus erythematosus pathway primarily reflected histone and chromatin-associated gene signatures rather than autoimmune mechanisms (Figures 1D,E). Collectively, these results suggest that the identified DEGs may contribute to ovarian cancer initiation, progression, and the regulation of its immune and stromal microenvironment.

### 3.2 Identification of key WGCNA modules and integrated determination of the core driver genes TRIP13 and CENPF

**Figure 2:**
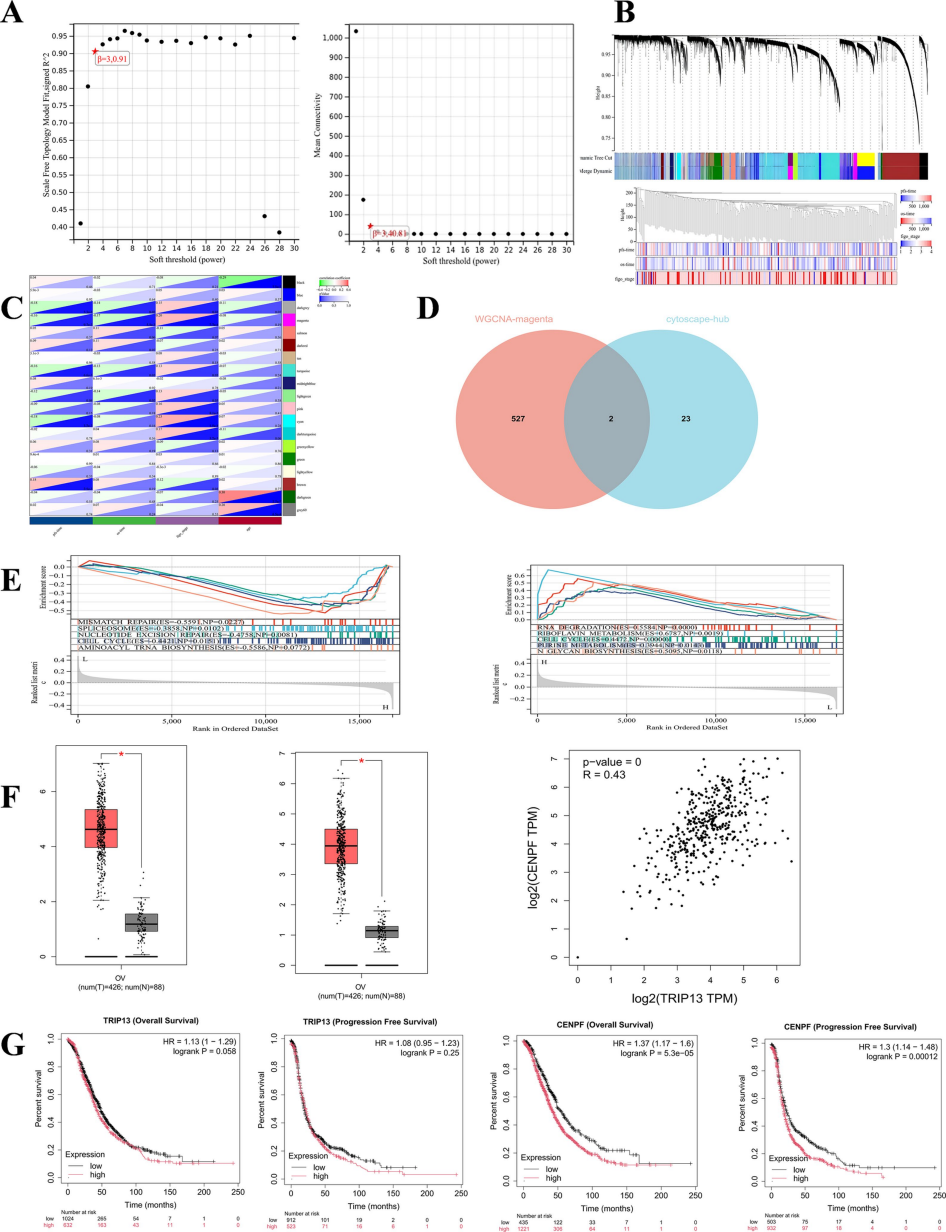
Identification of key WGCNA modules and integrated determination of the core driver genes TRIP13 and CENPF. **(A)** Soft-threshold selection for weighted gene co-expression network analysis (WGCNA): (Left) Scale-free topology model fit index; (Right) Mean connectivity. The optimal soft-thresholding power was determined to be 3 (*β* = 3). **(B)** WGCNA module construction and association with clinical traits: (Top) Dendrogram of gene co-expression modules identified from the GSE140082 dataset using WGCNA, resulting in 19 distinct modules; (Bottom) Module–trait relationship heatmap, depicting correlations between modules and clinical traits, including progression-free survival (PFS), overall survival (OS), and FIGO stage, with the magenta module showing a significant association. **(C)** Internal validation of the key module: Scatter plot illustrating the correlation between gene significance (with respect to clinical traits) and module membership within the magenta module, confirming high concordance between module genes and clinical phenotypes. **(D)** Core gene screening and overlap analysis: Venn diagram showing the intersection of genes from the WGCNA magenta module and protein–protein interaction (PPI) network analysis, identifying two overlapping key genes: TRIP13 and CENPF. **(E)** Biological functional enrichment analysis of key genes: Single-gene gene set enrichment analysis (GSEA) based on high- and low-expression groups, presenting the top five significantly enriched pathways for TRIP13 and CENPF. **(F)** Validation of key gene expression in the TCGA cohort: (Left) Comparative expression levels of TRIP13 and CENPF in serous ovarian cancer tumor tissues versus normal tissues; (Right) Correlation analysis between TRIP13 and CENPF transcript expression levels. **(G)** Prognostic analysis of candidate genes in the TCGA cohort. Survival curves for TRIP13 and CENPF generated via Kaplan–Meier plotter. CENPF high expression is significantly associated with poorer overall survival (HR = 1.37, 95% CI: 1.17–1.6, *p* = 5.3 × 10^−5^) and progression-free survival (HR = 1.30, 95% CI: 1.14–1.48, *p* = 0.00012). TRIP13 high expression shows a non-significant trend towards worse overall survival (HR = 1.13, 95% CI: 1–1.29, *p* = 0.058) and progression-free survival (HR = 1.08, 95% CI: 0.95–1.23, *p* = 0.25). HR, hazard ratio; *p*, log-rank *p*-value.

Weighted gene co-expression network analysis (WGCNA) of the GSE140082 dataset identified a robust scale-free topology at a soft-thresholding power of *β* = 3, supporting the reliability of the constructed network (Figure 2A). Nineteen co-expression modules were generated, among which the magenta module displayed the strongest correlations with critical clinical traits, including FIGO stage, progression-free survival (PFS), and overall survival (OS). These associations suggest that this module contains genes functionally linked to ovarian cancer progression. The module encompassed 529 genes, providing a focused set of biologically relevant candidates (Figures 2B,C).

By intersecting the genes within this clinically relevant module with 25 hub genes derived from the protein–protein interaction (PPI) network—identified using topological algorithms—TRIP13 and CENPF were determined to be the most representative driver genes (Figure 2D). Gene set enrichment analysis (GSEA) further demonstrated that their high-expression groups were significantly enriched in pathways governing cell-cycle control, DNA mismatch repair, GPI-anchor biosynthesis, and branched-chain amino acid metabolism, highlighting their involvement in proliferative and genomic maintenance processes essential for tumor progression (Figure 2E). Analysis of TCGA ovarian cancer data confirmed that both TRIP13 and CENPF were significantly overexpressed in tumor tissues relative to normal controls and exhibited a strong positive correlation, supporting their potential cooperative role in driving ovarian cancer initiation and malignant advancement (Figure 2F). To independently assess the prognostic potential of the identified hub genes, we analyzed their expression in the TCGA ovarian cancer cohort using the Kaplan–Meier plotter tool. This analysis revealed that CENPF high expression was significantly associated with poorer overall survival (HR = 1.37, 95% CI: 1.17–1.6, *p* = 5.3 × 10^−5^) and progression-free survival (HR = 1.30, 95% CI: 1.14–1.48, *p* = 0.00012). TRIP13 high expression showed a consistent, though non-significant, trend towards worse survival (OS: HR = 1.13, 95% CI: 1–1.29, *p* = 0.058; PFS: HR = 1.08, 95% CI: 0.95–1.23, *p* = 0.25). Given the limited direct clinical evidence for TRIP13 in ovarian cancer and its strong co-expression with disease traits in our WGCNA, we chose to focus our subsequent experimental validation on TRIP13 (Figure 2G).

### 3.3 Histological validation and prognostic significance of TRIP13

**Figure 3:**
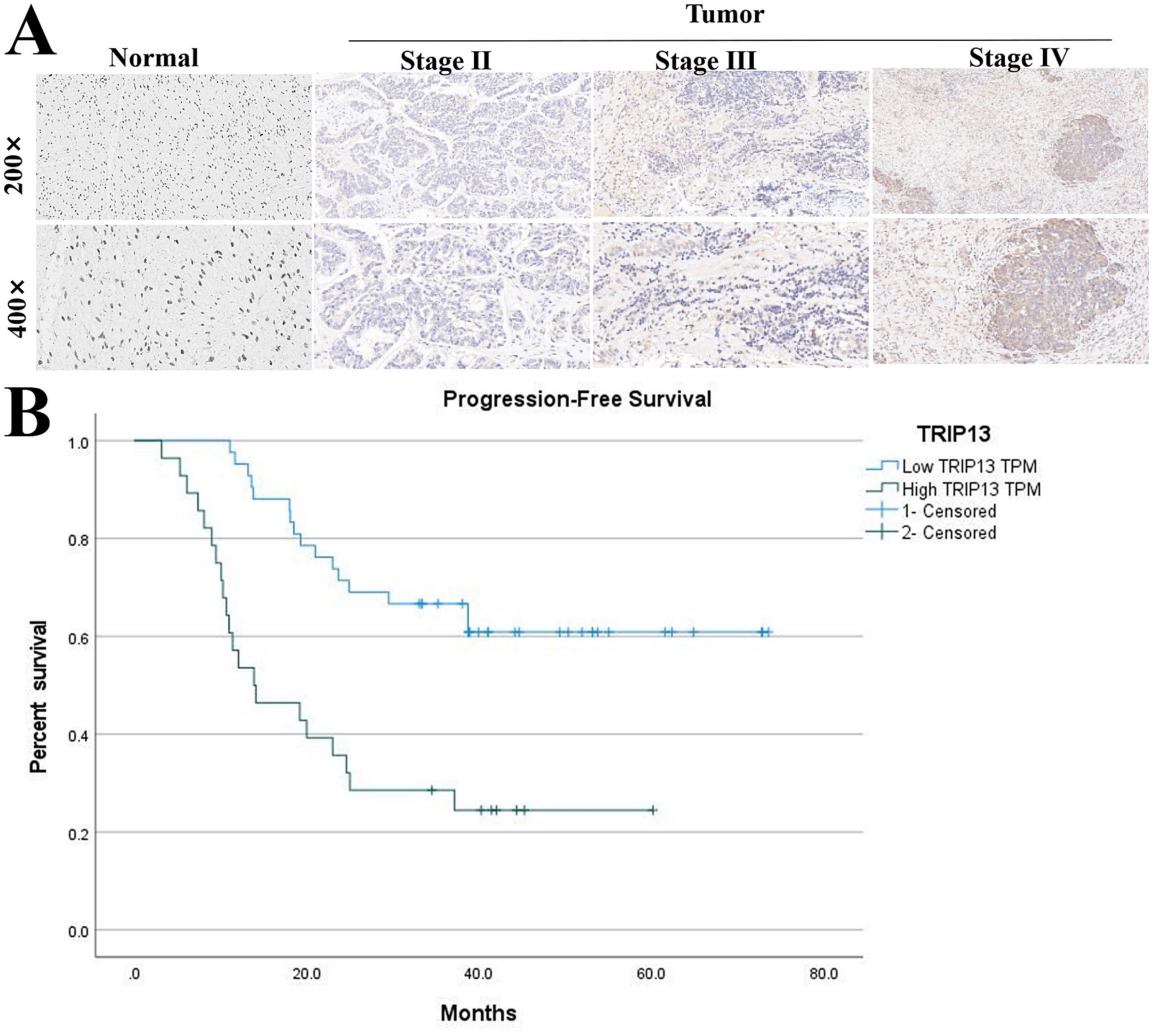
Histological validation and prognostic significance of TRIP13. **(A)** Representative immunohistochemical (IHC) staining images of TRIP13 protein in ovarian cancer and normal ovarian tissues. **(B)** Progression-free survival (PFS) analysis of ovarian cancer patients stratified by TRIP13 expression levels.

Immunohistochemical (IHC) assessment based on H-score quantification demonstrated that TRIP13 protein expression in ovarian cancer tissues was significantly and positively associated with FIGO stage, and its high-expression frequency was markedly increased compared with normal ovarian tissues (Tables 1, 2). These findings suggest that elevated TRIP13 expression may be involved in ovarian cancer progression (Figure 3A). Clinical follow-up analysis further revealed that high TRIP13 expression correlated with more advanced FIGO staging (*p* = 0.047) and reduced sensitivity to platinum-based chemotherapy (*p* = 0.015), as determined by *χ*^2^ and correlation analyses.

**Table 1:** Differential expression of TRIP13 protein in ovarian cancer and normal ovarian tissues and its statistical significance.

TRIP13	Tumor tissue		Normal ovarian tissue		*p*-value
Expression	Cases	Percentage	Cases	Percentage	
Low	42	60.00%	34	100%	*p* < 0.033
High	28	40.00%	0	0%	

**Table 2:** Correlation analysis between TRIP13 protein expression levels and clinical parameters in ovarian cancer patients.

Features	No. of patients	TRIP13		*p*-value
		Expression		
		Low	High	
All patients	70	42	28	
Age				0.26
<60 years	47	26	21	
≥60 years	23	16	7	
Stage				0.047
I	7	6	1	
II	14	10	4	
III	46	24	22	
IV	3	2	1	
Tumor size				0.435
<10 cm	29	19	10	
≥10 cm	41	23	18	
CA125				0.277
<600	43	28	15	
≥600	27	14	13	
Platinum reactivity				0.015
Sensitive	59	39	20	
Resistant	11	3	8	

In a cohort of 70 patients with high-grade serous ovarian carcinoma (HGSOC), the median progression-free survival (PFS) of the TRIP13 high-expression group was 13.9 months, whereas the low-expression group did not reach the median PFS threshold (50% progression) by the last follow-up. The survival difference between the two groups was statistically significant according to the log-rank test (*p* < 0.01). Multivariate Cox proportional hazards analysis identified TRIP13 high expression as an independent adverse prognostic factor for PFS (HR = 2.65, 95% CI: 1.28–5.51, *p* = 0.009), indicating that elevated TRIP13 expression corresponds to a substantially increased risk of disease progression (Figure 3B).

Overall, these results support the biological relevance of TRIP13 in ovarian cancer and suggest that it may serve as a potential biomarker for prognostic assessment and therapeutic stratification.

## 4 Discussion

Ovarian cancer is among the most lethal gynecological malignancies worldwide, with over 300,000 new cases diagnosed annually, and approximately 70% of patients presenting at advanced stages (11). Although surgery, chemotherapy, and maintenance therapy remain the mainstay of treatment, and immunotherapy and targeted therapy are emerging, the majority of patients ultimately experience relapse and develop platinum resistance, resulting in poor prognosis (12). Therefore, there is an urgent need to elucidate the pathogenesis of ovarian cancer and identify novel biomarkers and therapeutic targets. In this context, TRIP13 was found to be highly transcribed and overexpressed in ovarian cancer, positively correlating with FIGO stage and inversely associated with patient survival outcomes, suggesting its potential as a prognostic biomarker and therapeutic target.

Thyroid hormone receptor-interacting protein 13 (TRIP13) is a member of the AAA + ATPase family, which participates in diverse cellular processes including membrane transport, proteasome activity, DNA replication, and molecular motion (13). The human TRIP13 gene, located on chromosome 5, contains 14 exons encoding a 432-amino-acid protein (14). TRIP13 consists of a small N-terminal domain implicated in substrate recognition and a AAA + ATPase domain containing the ATP-binding site. Recent structural studies, including the TRIP13 hexameric complex with ligands, have provided insights into its functional mechanisms (15). TRIP13 plays a critical role in mitosis as a component of the spindle assembly checkpoint (SAC), maintaining genomic stability through the mitotic checkpoint complex (MCC), which includes BUBR1, BUB3, CDC20, and MAD2. TRIP13, via its ATPase activity, regulates the conformational state of MAD2, promoting checkpoint inactivation in cooperation with P31comet (16–18). Localization studies show TRIP13 resides at kinetochores and co-expresses with centromere/kinetochore components. Functional assays using mitotic markers such as MPM2 indicate that TRIP13 overexpression reduces mitotic arrest under spindle stress (19). Beyond mitosis, TRIP13 is involved in DNA double-strand break (DSB) repair during meiosis and mitotic cell cycles. It is essential for nonhomologous end-joining (NHEJ), contributing to genomic integrity during cell division (20–22). Aberrant TRIP13 expression has been observed in multiple malignancies, including lung, bladder, colorectal cancers, and multiple myeloma, often correlating with poor prognosis (23). Mechanistic studies reveal TRIP13 promotes tumor cell proliferation, migration, and invasion through pathways such as Akt/mTOR and Notch signaling (24). For example, TRIP13 downregulation in LUAD reduces phosphorylation of AKT, mTOR, P70S6K, and c-Myc, while TRIP13 knockdown in SKOV3 and OVCAR-3 cells suppresses Notch1 signaling and EOC cell proliferation/metastasis (25). Small-molecule TRIP13 inhibitors, developed based on its crystal structure, have shown efficacy in preclinical models (26).

In summary, TRIP13 functionally contributes to ovarian cancer tumorigenesis, proliferation, metastasis, and invasion. It is overexpressed in ovarian cancer tissues, with high expression levels associated with advanced FIGO stage and poor prognosis. It is noteworthy that our bioinformatic screening identified two strong candidates, TRIP13 and CENPF. Analysis of the TCGA cohort via Kaplan–Meier plotter confirmed the significant prognostic importance of CENPF (OS HR = 1.37, *p* = 5.3 × 10^−5^; PFS HR = 1.30, *p* = 0.00012), which is consistent with its established role in cell cycle regulation. However, we focused our experimental validation on TRIP13 for several reasons. First, its specific role and clinical relevance in ovarian cancer were less defined compared to CENPF. Second, TRIP13 showed a compelling, though non-significant, trend towards poor prognosis in the same TCGA data (OS HR = 1.13, *p* = 0.058; PFS HR = 1.08, *p* = 0.25), warranting further investigation. Most importantly, our subsequent clinical cohort validation provided robust evidence that high TRIP13 protein expression is an independent adverse prognostic factor for PFS (HR = 2.65, 95% CI: 1.28–5.51, *p* = 0.009), intimately linked to advanced stage and platinum resistance. This focused approach allowed us to generate novel, clinically actionable insights into TRIP13, establishing its value as a potential biomarker in ovarian cancer despite the stronger public database signal for its co-expressed partner, CENPF. The gene set enrichment analysis (GSEA) revealed that tumors with high TRIP13 expression were significantly enriched in pathways related to cell cycle regulation and DNA repair (Figure 2E). This finding provides a plausible mechanistic link to the aggressive clinical phenotype observed in our cohort. As a key regulator of the spindle assembly checkpoint (SAC) and DNA damage response, overexpression of TRIP13 could lead to premature checkpoint inactivation and compromised DNA repair fidelity, thereby promoting chromosomal instability. Such genomic instability is a hallmark of advanced and therapy-resistant tumors. We therefore hypothesize that TRIP13 overexpression confers a dual advantage to ovarian cancer cells: it drives uncontrolled proliferation through dysregulated cell cycle progression while simultaneously enhancing the cells’ ability to survive platinum-induced DNA damage. This mechanistic model directly explains the strong clinical correlations we observed between high TRIP13 expression, advanced FIGO stage, and platinum resistance. This study has several limitations. Firstly, the initial DEG screening relied on the GSE81778 dataset, which has a relatively small control group (*n* = 5), potentially affecting the stability of the identified gene list. Secondly, our clinical validation, though robust in its findings, is based on a single-center cohort of 70 HGSOC patients. The lack of an independent external cohort for validating the prognostic model limits the generalizability of our conclusions. Future studies involving larger, multi-center cohorts are warranted to confirm the clinical utility of TRIP13 as a biomarker. Additionally, the functional experiments were primarily correlative; further *in vitro* and *in vivo* studies are needed to definitively establish the causal role of TRIP13 in driving OC progression and chemoresistance. These findings suggest that TRIP13 may serve as a potential early biomarker and therapeutic target, highlighting its clinical relevance in ovarian cancer management.

## Data Availability

Publicly available datasets were analyzed in this study. This data can be found here: https://www.ncbi.nlm.nih.gov/geo/query/acc.cgi?acc=GSE140082; https://www.ncbi.nlm.nih.gov/geo/query/acc.cgi?acc=GSE81778.
